# Partial Functional Diversification of *Drosophila melanogaster* Septin Genes *Sep2* and *Sep5*

**DOI:** 10.1534/g3.116.028886

**Published:** 2016-05-02

**Authors:** Ryan S. O’Neill, Denise V. Clark

**Affiliations:** Department of Biology, University of New Brunswick, Fredericton, New Brunswick, E3B 5A3, Canada

**Keywords:** *Drosophila*, septin, oogenesis, gene duplication

## Abstract

The septin family of hetero-oligomeric complex-forming proteins can be divided into subgroups, and subgroup members are interchangeable at specific positions in the septin complex. *Drosophila melanogaster* has five septin genes, including the two SEPT6 subgroup members *Sep2* and *Sep5*. We previously found that *Sep2* has a unique function in oogenesis, which is not performed by *Sep5*. Here, we find that *Sep2* is uniquely required for follicle cell encapsulation of female germline cysts, and that *Sep2* and *Sep5* are redundant for follicle cell proliferation. The five *D. melanogaster* septins localize similarly in oogenesis, including as rings flanking the germline ring canals. Pnut fails to localize in *Sep5*; *Sep2* double mutant follicle cells, indicating that septin complexes fail to form in the absence of both Sep2 and Sep5. We also find that mutations in septins enhance the mutant phenotype of *bazooka*, a key component in the establishment of cell polarity, suggesting a link between septin function and cell polarity. Overall, this work suggests that *Sep5* has undergone partial loss of ancestral protein function, and demonstrates redundant and unique functions of septins.

Septins are a family of cytoskeletal GTP-binding proteins that form hetero-oligomeric rod-like complexes, which can further assemble into higher-order structures such as filaments or rings (John *et al.* 2007; Sirajuddin *et al.* 2007; Bertin *et al.* 2008; DeMay *et al.* 2011). Septins play roles in cell division (Hartwell *et al.* 1970; Longtine *et al.* 1996), regulation of cell shape and membrane rigidity (Tooley *et al.* 2009; Mostowy *et al.* 2011; Gilden *et al.* 2012), restriction of lateral diffusion at membranes (Schmidt and Nichols 2004; Caudron and Barral 2009; Hu *et al.* 2010; Kwitny *et al.* 2010; Spiliotis and Gladfelter 2012; Clay *et al.* 2014; Ewers *et al.* 2014), protein scaffolding (Hanrahan and Snyder 2003; Kozubowski *et al.* 2005; Kinoshita 2006; Hagiwara *et al.* 2011; Hall and Russell 2012; Feng *et al.* 2015), and maintenance of cell polarity (Barral *et al.* 2000; Takizawa *et al.* 2000; Spiliotis *et al.* 2008; Berepiki and Read 2013). Animal septins are divided into four subgroups: SEPT2, SEPT6, SEPT7, and SEPT3 (Kinoshita and Noda 2001). Septin hetero-oligomeric complexes have a subgroup-specific linear order (Kinoshita 2003; Sirajuddin *et al.* 2007, 2009; Bertin *et al.* 2008; Nakahira *et al.* 2010); for example, mammalian septin hexamers have a 7-6-2-2-6-7 subgroup organization. Septin subgroup members can be interchangeable (Kinoshita 2003), as observed for *Saccharomyces cerevisiae* septins Cdc11 and Shs1 (Bertin *et al.* 2008; Finnigan *et al.* 2015a,b) and mammalian septins SEPT6, SEPT8, and SEPT11 (Sellin *et al.* 2011a). However, subgroup members have distinct characteristics, such as protein interactions (Nakahira *et al.* 2010) and expression patterns (Cao *et al.* 2007; Tsang *et al.* 2008; Peterson and Petty 2010). The combination of interchangeability and distinct characteristics can lead to functionally distinct populations of septin complexes acting within cells and across tissues (Hernández-Rodríguez *et al.* 2014). Duplication and functional divergence of septin genes was likely important for generating functional diversity in the septin gene family. 

Whereas mammals have 13 septin genes (Cao *et al.* 2007), *Drosophila melanogaster* has five (Adam *et al.* 2000): *Sep1* and *Sep4* (SEPT2 subgroup), *Sep2* and *Sep5* (SEPT6), and *pnut* (SEPT7). *D. melanogaster* septin complexes with Sep1, Sep2, and Pnut have been isolated (Field *et al.* 1996; Oegema *et al.* 1998). Protein–protein interaction data indicate that Sep5 also interacts with Sep1 and Pnut (Guruharsha *et al.* 2011), suggesting interchangeability of Sep2 and Sep5. Whereas *Sep2* and *Sep5* single mutants survive to adulthood, *Sep5*; *Sep2* double mutants lack imaginal discs and die as prepupae (O’Neill and Clark 2013), similar to *pnut* mutants (Neufeld and Rubin 1994). *Sep2* mutants also have oogenesis defects that are not rescued by overexpression of *Sep5*, showing that Sep2 and Sep5 have diverged in function (O’Neill and Clark 2013). 

Here, we further explore *Sep2* and *Sep5* in gametogenesis, finding that *Sep2* has a unique function for follicle cell encapsulation of female germline cysts, and is redundant with *Sep5* for follicle cell proliferation and localization of Pnut. Further, Sep2 and Sep5 have similar subcellular localization in oogenesis. Heterozygosity for mutations in *Sep2*, *Sep5*, and *pnut* enhance the embryonic lethal phenotype of *bazooka*, a key regulator of epithelial cell polarity. Although several components of cell polarity are not perturbed in septin mutants, the interaction with *bazooka* suggests a connection between cell polarity and septin function in *D. melanogaster*. This work highlights the complexity of septin function in multicellular organisms, where septin subgroup members can have redundant functions as well as unique functions required in certain contexts. 

## Materials and Methods

### Fly strains and culture

w^1118^, y^1^ w*; P{Sep2-GFP.SG}3, w*; P{UASp-Sep1.GFP}3, w*; P{UASp-Sep4.GFP}3/TM3 Sb^1^, w*; P{UASp-Sep5.GFP}3, *w^1118^; P{GAL4::VP16-nos.UTR}CG6325^MVD1^*, y^1^ w*; P{Act5C-GAL4}25FO1/CyO, y^1^ w*; P{tubP-GAL4}LL7/TM3 Sb^1^ Ser^1^, *y^1^ baz^4^/FM7a*, *pnut^XP^/T(2;3)SM6a-TM6B Tb^1^*, P{hsFLP}22, w*, *w^1118^; P{neoFRT}82BP{Ubi-GFP(S65T)nls}3R/TM6B Tb^1^*, *P{neoFRT}82B* *cu^1^sr^1^e^s^ca^1^*, and y^1^ w* P{PTT-GC}baz^CC01941^ were obtained from Bloomington *Drosophila* Stock Center at Indiana University. *Sep2^2^*, *Sep5^2^*, *P{UASp-Sep2}18A*, and *P{UASp-Sep5}33B* were generated as described in O’Neill and Clark (2013). Flies were reared on standard cornmeal-molasses-agar media or Equation 4-24 plain instant media (Carolina Biological Supply Company, Burlington, NC) at 25° and 60% relative humidity. Crosses to generate mitotic clones for eye, female germline, and egg length analyses were between w* hsFLP; Sep5^2^; FRT P{Ubi-GFP, w^+Mc^} virgin females and w*; Sep5^2^; FRT cu^1^ sr^1^ e^s^ ca^1^, w*; FRT cu^1^ Sep2^2^/TM6B, or w*; Sep5^2^; FRT cu^1^ Sep2^2^/TM6B males. Mitotic clones were induced by heat shocking larvae at 38°. Clones in eyes were generated by a 1 hr heat shock at the second instar. Areas of mitotic clone twin spots were measured from stereomicroscope digital images using Fiji (Schindelin *et al.* 2013). Clones for analyses of egg length and ovary phenotypes were generated by 1 hr heat shocks 3 and 4 d in a row, respectively, starting at the second instar. 

### Immunofluorescence

One-day-old females were aged for 2 d with yeast paste and males. Ovaries were dissected on ice in PBS (phosphate buffered saline) and fixed for 20 min in 4% w/v paraformaldehyde in PBS. Fixed samples were washed in PBST (PBS with 0.1% Triton-X-100) and then permeabilized for 2 hr in PBS with 1% Triton-X-100 and 2% normal goat serum (NGS; Jackson ImmunoResearch Laboratories Inc.). Samples were washed in PBST + 2% NGS and then incubated with primary antibody at 4° overnight. Samples were washed six times in PBST + 2% NGS and then incubated for 2 hr with secondary antibody. Samples were washed with PBST and then incubated with 10 µM Draq5 (Cell Signaling Technology) and 10 µg/mL rhodamine phalloidin (Sigma-Aldrich) for 20 min. Samples were finally washed in PBS and stored in 90% glycerol:PBS at 4°. The following primary antibody concentrates were obtained from the Developmental Hybridoma Studies Bank at The University of Iowa: anti-Orb 6H4 (diluted 1:20), anti-Pnut 4C9H4 (1:20), anti-α-spectrin 3A9 (1:100), anti-Hts-RC (1:100), anti-Discs large 4F3 (1:20), and anti-Armadillo (1:20). Anti-Anillin (1:50) was a gift from Julie Brill. The secondary antibodies Alexa Fluor 594-conjugated AffiniPure Goat Anti-Mouse IgG and Alexa Fluor 488-conjugated AffiniPure Goat Anti-Rabbit IgG (H + L) were purchased from Jackson ImmunoResearch Laboratories Inc. (diluted 1:500). Fluorescence images were acquired using a Leica SP2 Confocal microscope. Fiji (Schindelin *et al.* 2013) was used for image processing. 

### Male fertility

Males of various genotypes and wild-type (*w^1118^*) virgin females were collected daily and separately aged for 3–4 d with yeast paste. Individual males and five virgin females were placed in single vials. After 5 d, males that produced larvae were scored as fertile. 

### Septin enhancement of baz^4^

We tested for enhancement of the *baz^4^* embryonic lethal defective cuticle phenotype similarly to Shao *et al.* (2010). Virgin females of genotypes baz^4^/w*; *Sep2^2^/Sep2^+^*, baz^4^/w*; *Sep5^2^/Sep5^+^*, baz^4^/w*; *pnut^XP^/pnut^+^*, and baz^4^/w* were crossed to *w^1118^* males. Eggs were collected for 24 hr on grape agar plates spread with yeast paste, and then were aged for 48 hr. Of the *baz^4^/Y* arrested embryos, half were heterozygous for a mutant septin allele and half were homozygous wild type, except for the control where all were homozygous wild type. Unhatched eggs were dechorionated in 50% bleach for 2–3 min, mounted on slides with 1:1 Hoyer’s Mountant:lactic acid, and baked at 60° overnight. Embryonic cuticles were scored blind into six categories using a Leica Digital Light Microscope with 40 × objective. 

Fly strains are available upon request. Supplemental Material, Figure S1 shows *Sep5^2^*; *Sep2^2^* germline cysts have wild-type distribution of several proteins, Figure S2 shows *Sep2-GFP* and *Sep5-GFP* fusion proteins are functional, Figure S3 shows Sep1-GFP and Sep4-GFP localization in oogenesis, and Figure S4 shows *Sep5^2^*; *Sep2^2^* follicle cells have wild-type distribution of several cell polarity proteins.

### Data availability

The authors state that all data necessary for confirming the conclusions presented in the article are represented fully within the article.

## Results

### Sep2 is required for follicle cell encapsulation of germline cysts

In *Drosophila* oogenesis (reviewed in Spradling 1993), a germline stem cell in the germarium produces a daughter cystoblast that undergoes four rounds of incomplete division to produce a cyst of 16 cells with cytoplasm connected by stable ring canals. One of the 16 cells becomes the oocyte and the other 15 become nurse cells. Precursor follicle cells envelope the germline cyst to form an egg chamber (Horne-Badovinac and Bilder 2005). Follicle cells proliferate in the germarium and early egg chambers. The egg chamber then exits the germarium and travels posteriorly along the ovariole as it develops into a mature egg. 

**Figure 1:**
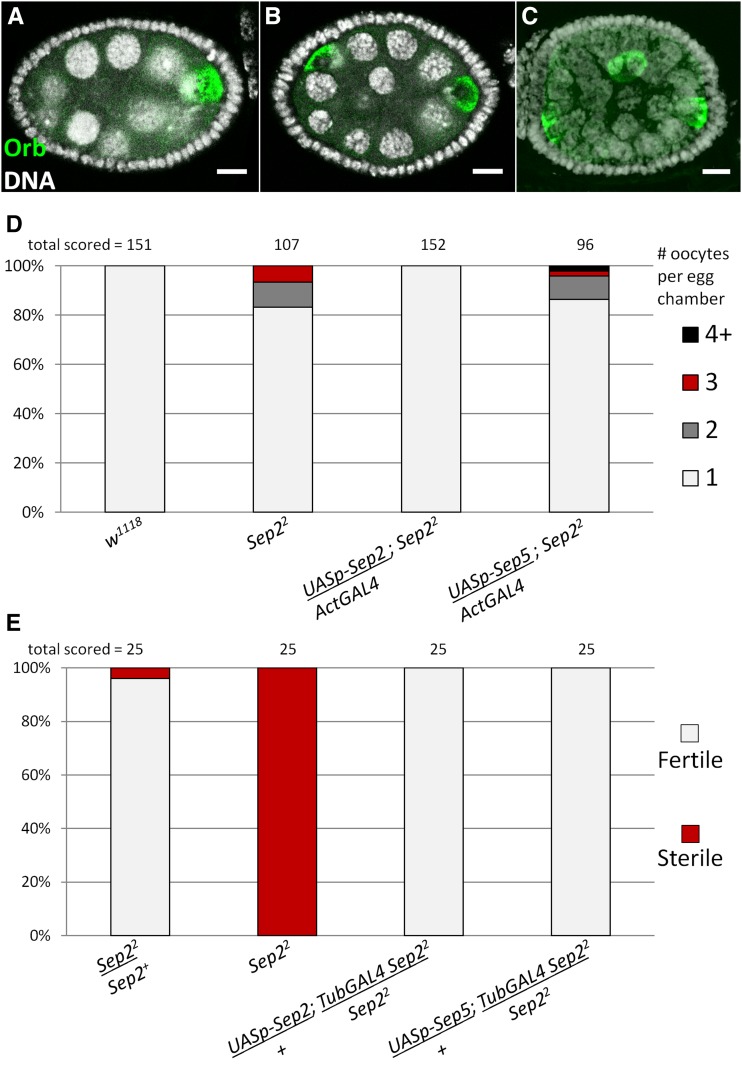
*Sep2* has a unique function in follicle cell encapsulation. Wild-type egg chambers contain a single cyst with 15 nurse cells and one oocyte (A), whereas some *Sep2^2^* egg chambers have multiple cysts, for example with 30 nurse cells and two oocytes (B) or 45 nurse cells and three oocytes (C). (D) Stage six to eight egg chambers of various genotypes were scored for number of cysts. 100% of wild-type egg chambers have a single oocyte, whereas ∼17% of *Sep2^2^* egg chambers contain multiple cysts. Using *Act5C-GAL4* to express *P{UASp-Sep2}18A*, a *Sep2* cDNA transgene, rescues this *Sep2^2^* phenotype, but expressing *P{UASp-Sep5}33B*, a *Sep5* cDNA transgene, does not rescue. (E) *Sep2^2^* males are sterile; however, either a *Sep2* or *Sep5* cDNA transgene driven by *tubP-GAL4* rescues *Sep2^2^*. For each genotype, the total number of egg chambers or individual males scored is shown above each column. Scale bar = 10 µm. cDNA, complementary DNA; Sep, septin.

We previously found that *Sep2^2^* mutants, which contain a large deletion of the *Sep2* coding region and are thus expected to be null mutants, have egg chambers with abnormal numbers of nurse cells (O’Neill and Clark 2013). To determine if the *Sep2^2^* egg chamber defects are due to abnormal cystoblast divisions or fusion of multiple cysts into a single egg chamber, we counted oocytes and nurse cells per egg chamber by staining ovaries for Orb, which accumulates in the oocyte (Lantz *et al.* 1994), and DNA, which highlights polytene nurse cell nuclei (Dej and Spradling 1999; Figure 1). Whereas wild-type egg chambers always contain a single cyst (*i.e.*, one oocyte and 15 nurse cells), 17% (18/107) of *Sep2^2^* egg chambers contain multiple cysts (*e.g.*, two oocytes and 30 nurse cells) and 2% (2/107) have numbers of nurse cells indicating failure of cystoblast division (*i.e.*, not a multiple of 15). The *Sep2^2^* egg chamber phenotype was rescued by driving expression of a *Sep2* cDNA transgene, but not a *Sep5* cDNA transgene, using *Act5C-GAL4*, consistent with previous results (O’Neill and Clark 2013). We also examined male fertility and found that *Sep2^2^* males are sterile; however, *Sep2^2^* male sterility is rescued by driving expression of either a *Sep2* or *Sep5* transgene using *tubP-GAL4* (Figure 1E). 

### Sep2 and Sep5 are redundant for follicle cell proliferation

**Figure 2:**
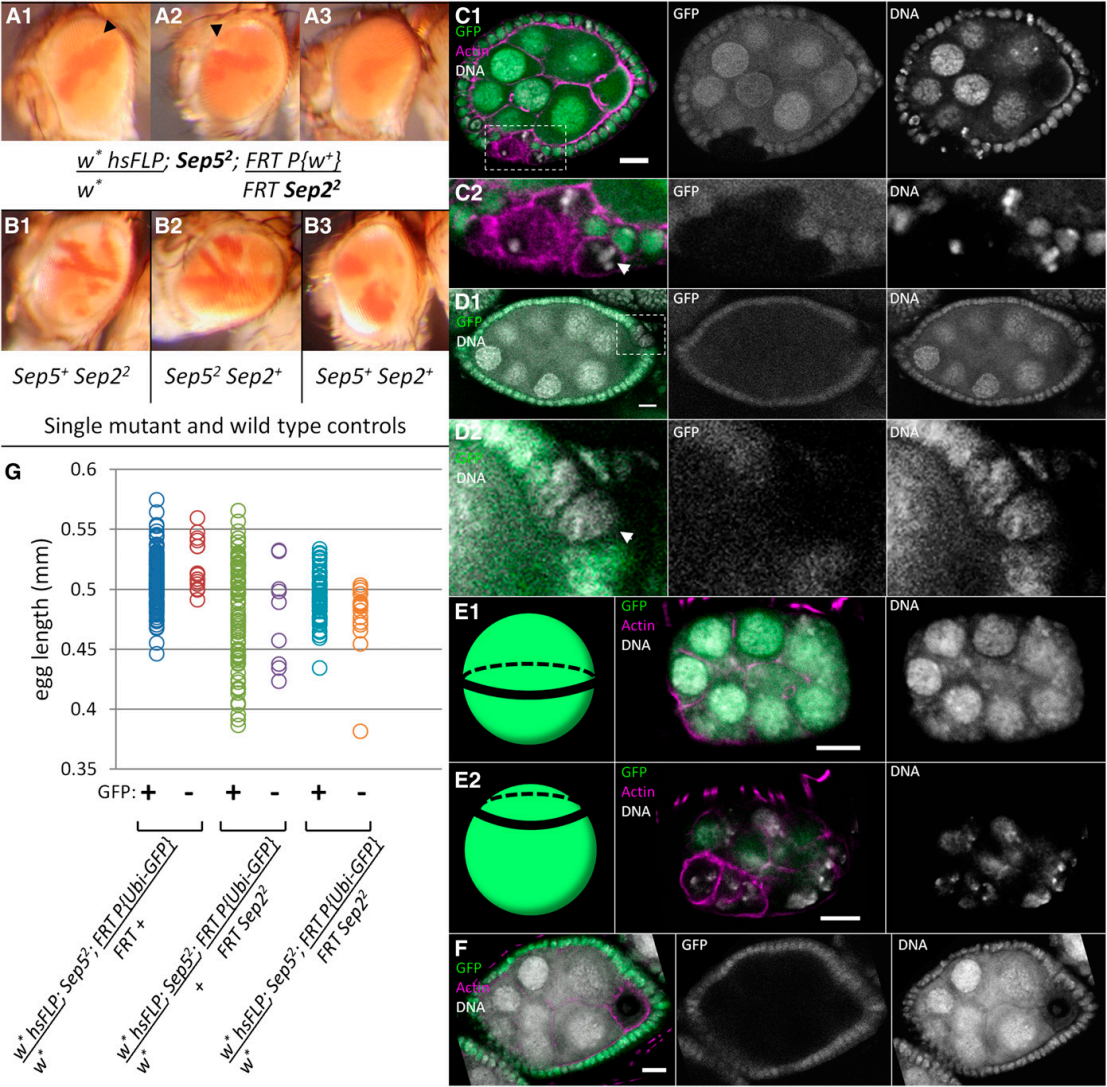
*Sep2* and *Sep5* share a redundant function for follicle cell proliferation but are dispensable for cystoblast divisions. w* hsFLP; Sep5^2^/Sep5^+^; FRT P{Ubi-GFP}/FRT cu^1^ sr^1^ e^s^ ca^1^, w* hsFLP; Sep5^2^; FRT P{Ubi-GFP}/FRT cu^1^ sr^1^ e^s^ ca^1^, w* hsFLP; Sep5^2^/Sep5^+^; FRT P{Ubi-GFP}/FRT cu^1^ Sep2^2^, and w* hsFLP; Sep5^2^; FRT P{Ubi-GFP}/FRT cu^1^ Sep2^2^ were used to generate wild-type control, wild-type in a *Sep5^2^* background, *Sep2* single mutant, and *Sep5; Sep2* double mutant clones, respectively. (A) *Sep5^2^; Sep2^2^* mitotic clones are small (A1, A2, arrowheads) or absent (A3) in eyes. Dark orange clones in a light orange background are the homozygous *Sep2^+^* twin spots of *Sep2^2^* clones that did not grow in a *Sep5^2^* background. (B) Dark orange:white area ratios of *Sep2^2^* clones in a *Sep5^+^* background (B1), and *Sep2^+^* clones in *Sep5^2^/Sep5^+^* (B2) and *Sep5^+^* (B3) backgrounds are not significantly different. (C) *Sep5^2^; Sep2^2^* follicle cells are often misshapen, have pyknotic nuclei (arrowhead), and often fail to maintain epithelial structure (*e.g.*, stage six egg chamber shown in C1). (D) Some *Sep5^2^; Sep2^2^* follicle cells have enlarged nuclei (*e.g.*, stage eight egg chamber in D1, arrowhead in D2), indicating failure of cytokinesis. (E) Egg chambers with only *Sep5^2^; Sep2^2^* follicle cells have severely reduced follicle cell numbers (E1 and E2 show focal planes through the middle and follicle cell layer of the same stage four egg chamber, respectively). (F) *Sep5^2^; Sep2^2^* germline clones appear wild-type. (G) Genotypes of laid eggs were determined by presence of GFP (GFP negative eggs arose from either wild-type, *Sep2^2^* or *Sep5^2^; Sep2^2^* germline clones). Reduction in length of eggs from w* hsFLP; Sep5^2^/Sep5^+^; FRT P{Ubi-GFP}/FRT cu^1^ Sep2^2^ females is independent of the genotype of germline clone genotype, suggesting that follicle cell genotype determines egg length. Scale bar = 10 µm. GFP, green fluorescent protein; Sep, septin.

*Sep5^2^* mutants, which have a deletion of the entire *Sep5* coding region, are viable and fertile; however, *Sep5*; *Sep2* double mutants lack imaginal discs and arrest as prepupae (O’Neill and Clark 2013). We used mitotic clones to investigate *Sep5^2^*; *Sep2^2^* adult structures. In eyes, *Sep5^2^*; *Sep2^2^* mitotic clones (Figure 2A) fail to proliferate compared to single mutant and wild-type control clones (Figure 2B). Mitotic clone twin spot area ratios (dark orange:white) among the three Figure 2B genotypes, w* hsFLP; *Sep5^2^/Sep5^+^*; *FRT P{Ubi-GFP w^+^}/FRT cu^1^Sep2^2^* (mean ratio 1.34, N = 40;), w* hsFLP; *Sep5^2^*; *FRT P{Ubi-GFP w^+^}/FRT cu^1^ sr^1^ e^s^ ca^1^* (mean ratio 1.33, N = 31), and w* hsFLP; *Sep5^2^/Sep5^+^*; *FRT P{Ubi-GFP w^+^}/FRT cu^1^ sr^1^ e^s^ ca^1^* (mean ratio 1.43, N = 42), were not significantly different (one-way ANOVA, p-value = 0.41). The curvature of the eye and transparency of white ommatidia likely made the edges of white clones appear pigmented, thereby biasing the ratios of twin spot areas toward dark orange. In contrast, only 12 of 24 dark orange clones in w* hsFLP; *Sep5^2^*; *FRT P{Ubi-GFP w^+^}/FRT cu^1^Sep2^2^* had a white mutant twin spot, and the mean dark orange:white ratio was 18. Therefore, *Sep5^2^*; *Sep2^2^* mutant clones have a growth disadvantage (Figure 2A). 

In oogenesis, double mutant follicle cell clones in w* hsFLP; *Sep5^2^*; *FRT P{Ubi-GFP}/FRT cu^1^Sep2^2^* were less common and usually contained < 10 cells, compared to control clones in w* hsFLP; *Sep5^2^*; *FRT P{Ubi-GFP}/FRT cu^1^ sr^1^ e^s^ ca^1^* and w* hsFLP; *Sep5^2^/Sep5^+^*; *FRT P{Ubi-GFP}/FRT cu^1^Sep2^2^*, which were more common and always consisted of more than 10 cells (Table 1). The majority of *Sep5^2^*; *Sep2^2^* follicle cell clones had some cells with pyknotic nuclei (Figure 2C) indicating cell death, enlarged nuclei (Figure 2D) indicating failure of cytokinesis, and epithelial defects where cells had lost their place in the follicular epithelium (Figure 2C and Table 1). Some *Sep2^2^* follicle cell clones in a *Sep5^2^/Sep5^+^* background also had enlarged nuclei (Table 1). Rare ovarioles were observed that had entirely *Sep5^2^*; *Sep2^2^* follicle cells; the few follicle cells surrounding cysts in these ovarioles were misshapen and appeared to have pyknotic nuclei and lack cell polarity, and these egg chambers were never observed beyond stage 4 (Figure 2E). In contrast, *Sep5^2^*; *Sep2^2^* germline clones were relatively common and did not have defects in cell number (Figure 2F). *Sep5^2^*; *Sep2^2^* germline clones have wild-type staining for Orb in the oocyte, α-spectrin at the fusome, actin and Hts-RC at the ring canals, and anillin at the cytokinetic furrow (Figure S1). 

**Table 1:** Mosaic egg chamber phenotypes

			Follicle Cell Phenotypes*^a^*			
Genotype	GFP −ve Germline	GFP −ve Follicle Cells	Small Clones*^b^*	Pyknotic Nuclei	Epithelial Defects*^c^*	Large Nuclei
*Sep5^2^* control	0.27 (8/30)	0.8 (24/30)	0	0	0.04 (1/24)	0
*Sep2^2^* control	0.26 (8/31)	0.68 (21/31)	0	0	0.05 (1/21)	0.19 (4/21)
*Sep5^2^*; *Sep2^2^*	0.21 (11/52)	0.38 (20/52)	0.85 (17/20)	0.75 (15/20)	0.7 (14/20)	0.65 (13/20)

Stage seven to eight mosaic egg chambers from three genotypes were scored. *Sep5^2^* control was w* hsFLP; Sep5^2^; FRT P{Ubi-GFP}/FRT cu^1^ sr^1^ e^s^ ca^1^. *Sep2^2^* control was w* hsFLP; Sep5^2^/Sep5^+^; FRT P{Ubi-GFP}/FRT cu^1^ Sep2^2^. *Sep5^2^; Sep2^2^* was w* hsFLP; Sep5^2^; FRT P{Ubi-GFP}/FRT cu^1^ Sep2^2^.

a Showing proportion of egg chambers with GFP negative follicle cells that had at least one GFP negative follicle cell with a given phenotype.

b GFP negative follicle cell clones with < 10 cells.

c GFP negative follicle cells that were either outside the follicle cell epithelium or inside the space occupied by the germline.

We previously observed that *Sep2^2^* eggs were shorter than wild-type eggs (O’Neill and Clark 2013), similar to *pnut^XP^* eggs (Adam *et al.* 2000) and a small egg or dumpless phenotype (Spradling 1993). To determine if abnormal egg morphology is due to loss of septins in the maternal germline, we induced mitotic recombination in *hsFLP*; *Sep5^2^*; *FRT P{Ubi-GFP}/FRT cu^1^ sr^1^ e^s^ ca^1^*, *hsFLP*; *Sep5^2^/Sep5^+^*; *FRT P{Ubi-GFP}/FRT cu^1^Sep2^2^*, and *hsFLP*; *Sep5^2^*; *FRT P{Ubi-GFP}/FRT cu^1^Sep2^2^* females mated to Oregon-R males, and looked for a correlation between egg length and the presence of GFP (Figure 2G). In *Sep2^2^* mosaic flies, egg length is independent of GFP; therefore, we inferred that the genotype of the follicle cells (which are presumably mosaic) determines egg morphology in *Sep2^2^* mutants. *Sep5^2^*; *Sep2^2^* mosaic flies have fewer short eggs compared to *Sep2^2^* mosaics, consistent with the failure of proliferation in *Sep5^2^*; *Sep2^2^* follicle cell clones (Figure 2, C and D). 

### Septin localization in oogenesis

We tested whether *Sep2-GFP*, which contains upstream and downstream noncoding sequences of *Sep2* to drive its expression (Silverman-Gavrila *et al.* 2008), and *Sep5-GFP*, which has a UAS and therefore requires GAL4 for expression (Su *et al.* 2013), encode functional proteins by performing rescue experiments (Figure S2). *Sep2-GFP* rescues the *Sep2^2^* nurse cell phenotype. A few (3.5%, 4/113) *Sep2-GFP Sep2^2^/Sep2^+^* egg chambers had only 13–14 nurse cells, which could indicate that Sep2-GFP has a weak dominant negative effect on cystoblast divisions. Both *Sep2-GFP* and *Act5C-GAL4 > Sep5-GFP* rescue the *Sep5^2^*; *Sep2^2^* prepupal lethal phenotype (note that double mutants expressing *Act5C-GAL4 > Sep5-GFP* arrest at the end of metamorphosis, but this is attributed to GAL4 sensitivity because *Sep2-GFP* transgenics with GAL4 also arrest). Two copies of *Sep2-GFP* increase lethality at the end of metamorphosis, further suggesting a weak dominant negative effect of the transgene. Together, these results indicate that the *Sep2-GFP* and *Sep5-GFP* transgenes encode functional proteins and should therefore be useful for investigating Sep2 and Sep5 localization. 

**Figure 3:**
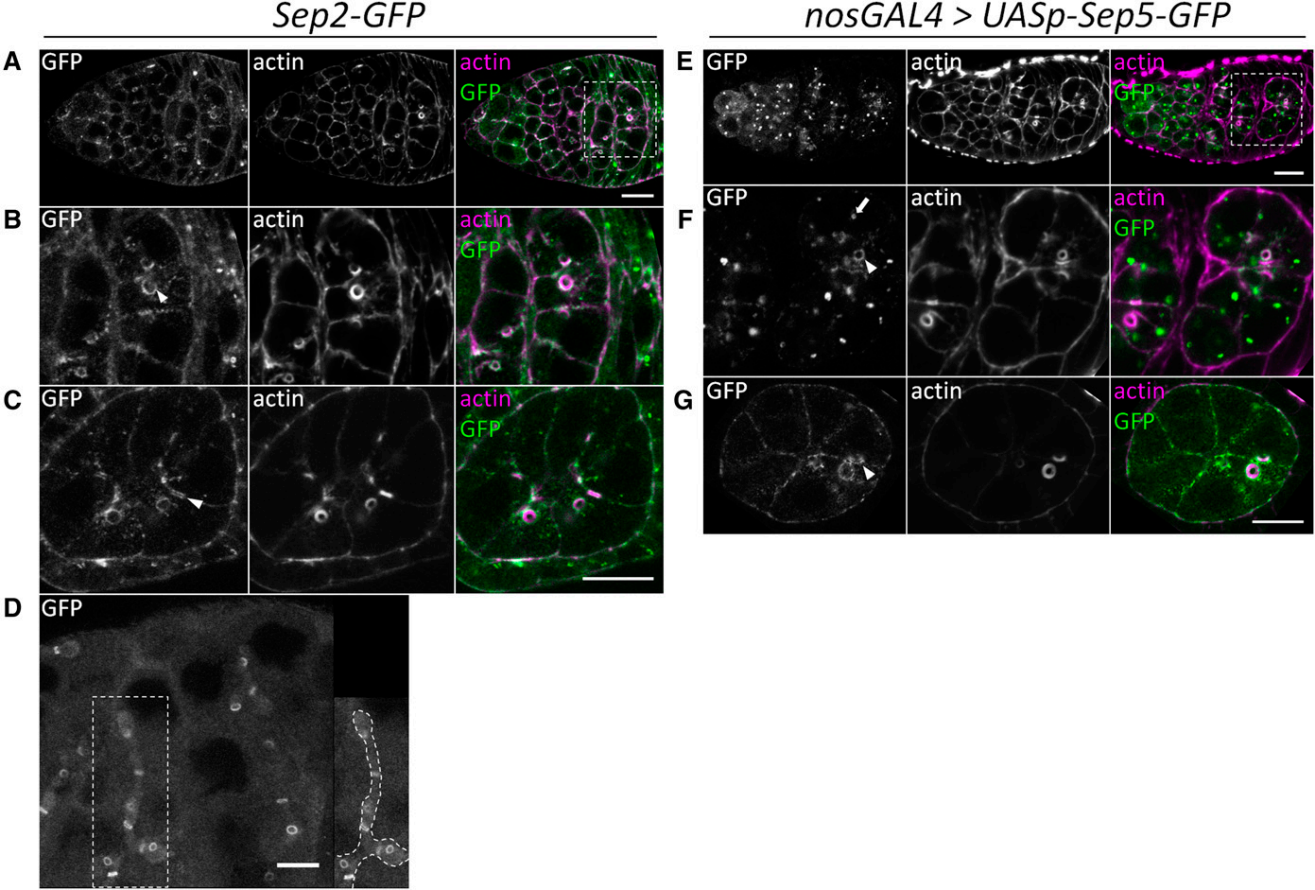
Sep2-GFP and Sep5-GFP localize similarly in oogenesis. *Sep2-GFP* (A–D) and *nosGAL4 > UASp-Sep5-GFP* (E–G) were used to characterize Sep2 and Sep5 localization. In the germarium (A, B, E, and F) and egg chambers (*e.g.*, stage six egg chambers in C and G), Sep2-GFP and Sep5-GFP localize cytoplasmically with a concentration at the cell cortex, and are concentrated at the outer rim of ring canals (B, C, F, and G, arrowheads) and as cytoplasmic puncta (F, arrow). Beginning at around stage four of egg chamber development, septin-GFPs appear to form rings that flank either side of the ring canal (C and G, arrowheads). Sep2-GFP is localized to the cell cortex of follicle cells (C). In testes (D), Sep2-GFP has a cytoplasmic localization, is localized at ring canals (in some cases as double rings), and appears to localize to the fusome (for example, structure enclosed in large white box, and highlighted with a dotted outline in right panel). B and F show magnifications of white boxes in A and E, respectively. Scale bar = 10 µm. GFP, green fluorescent protein; Sep, septin.

**Figure 4:**
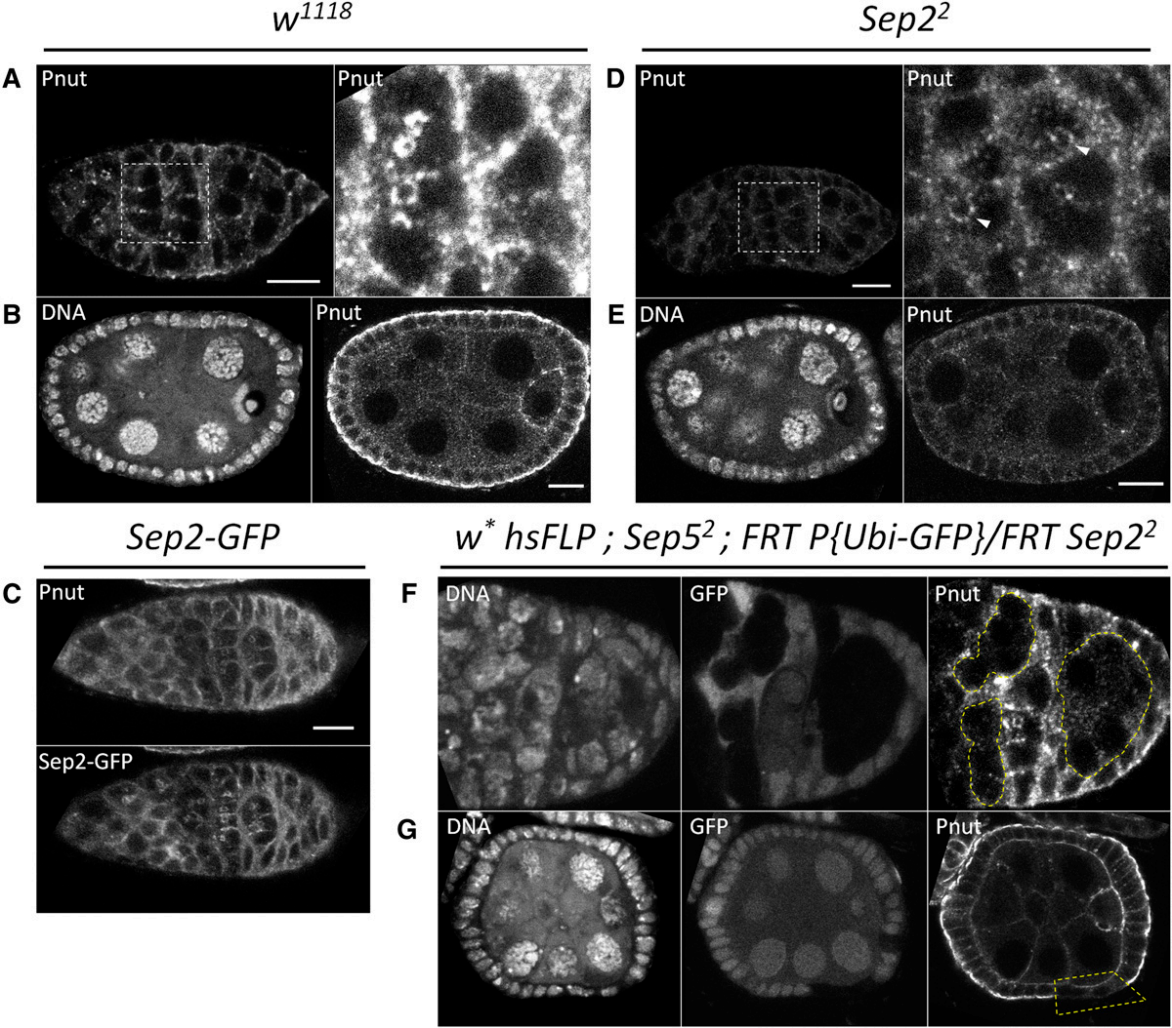
*Sep2* and *Sep5* are redundant for Pnut localization. In wild-type (*w^1118^*), Pnut localizes cytoplasmically and at ring canals in the germarium (A, with magnification of white box in right panel) and later egg chambers (not shown). In follicle cells of later stage egg chambers (*e.g.*, stage six egg chamber in B), Pnut is most concentrated basally. Pnut colocalizes with Sep2-GFP (C). Pnut is present cytoplasmically and at ring canals in *Sep2^2^* mutants (D, with magnification of white box in right panel with arrowheads indicating ring canal staining), and in egg chamber follicle cells (*e.g.*, stage six egg chamber in E), although it is less concentrated compared to *w^1118^* (A and B). Compared to GFP positive cells, which appear to have wild-type Pnut, *Sep5^2^*; *Sep2^2^* cells (GFP negative, highlighted with yellow lines) lack wild-type Pnut localization; in particular, Pnut still has some cytoplasmic signal, but fails to localize to ring canals (F) and the basal side of follicle cells (G) in *Sep5^2^*; *Sep2^2^* double mutant cells. All images were collected and processed identically. Scale bar = 10 µm. GFP, green fluorescent protein; Sep, septin.

We characterized the localization of Sep2-GFP and Sep5-GFP (Figure 3), as well as Sep1-GFP and Sep4-GFP (Figure S3), and Pnut immunostaining (Figure 4), in oogenesis, finding that they localize similarly. Note that, except for *Sep2-GFP* which contains its own promoter and regulatory region, the septin-GFP transgenes were expressed using *nos-GAL4*; although RNA-sequencing of whole ovaries shows that *Sep5*, *Sep1*, and *Sep4* are expressed (modENCODE tissue RNA-seq presented on FlyBase.org; Attrill *et al.* 2016), their expression patterns in ovaries are unknown. In proliferating germline cells, septin-GFPs are localized cytoplasmically, with a higher concentration at the cell cortex and cytokinetic furrows (Figure 3, B and F). They remain at the outer rim of ring canals throughout oogenesis, appearing as double rings flanking the germline ring canals from around stages four to nine of egg chamber development (Figure 3, C and G). At around stage four of egg chamber development, Sep2-GFP and Sep5-GFP localize as double rings flanking the germline ring canals (Figure 3, C and G), indicating that they do not form a single continuous structure that spans the cell–cell boundary. Only a single ring is observed after egg chamber expansion around stage nine (not shown). We observed these double rings in unfixed *Sep2-GFP* egg chambers, showing that they are not an artifact of fixation (data not shown). In spermatogenesis, Sep2-GFP also localizes to germline ring canals, occasionally appearing as double rings, and also appears to localize to the fusome (Figure 3E). Septin-GFPs, particularly those driven by *nosGAL4*, also localize as cytoplasmic puncta and rings in both germline and follicle cells throughout oogenesis (example Figure 3F). Sep2-GFP localizes to the cortex of follicle cells in oogenesis (Figure 3C). After stage ten, Sep2-GFP is more strongly concentrated at the cortex of the oocyte relative to nurse cells, and is strongly expressed in border cells (not shown), as found for Sep1 (Fares *et al.* 1995).

Pnut localization in oogenesis, which was previously described by Adam (1999), is similar to that of the septin-GFPs (Figure 4, A and B) and colocalizes with Sep2-GFP (Figure 4C); however, Pnut differs from Sep2-GFP in that it is localized more strongly to the basal cortex compared to the lateral cortex in follicle cells (Figure 4B). In *Sep2^2^* mutants, Pnut is less concentrated in the germarium, although it still weakly localizes to ring canals (Figure 4D) and in older egg chambers (Figure 4E). However, in *Sep5^2^*; *Sep2^2^* clones, Pnut fails to localize to ring canals of germline cysts (Figure 4F) or in follicle cells (Figure 4G). *Sep5^2^* mutant cells appear to have wild-type Pnut (compare GFP-positive cells in Figure 4, G and B). Therefore, *Sep2* and *Sep5* are redundant for Pnut localization at female germline ring canals and the cortex of follicle cells.

### Sep2^2^, Sep5^2^, and pnut^XP^ enhance baz^4^ defective embryonic cuticle phenotype

**Figure 5:**
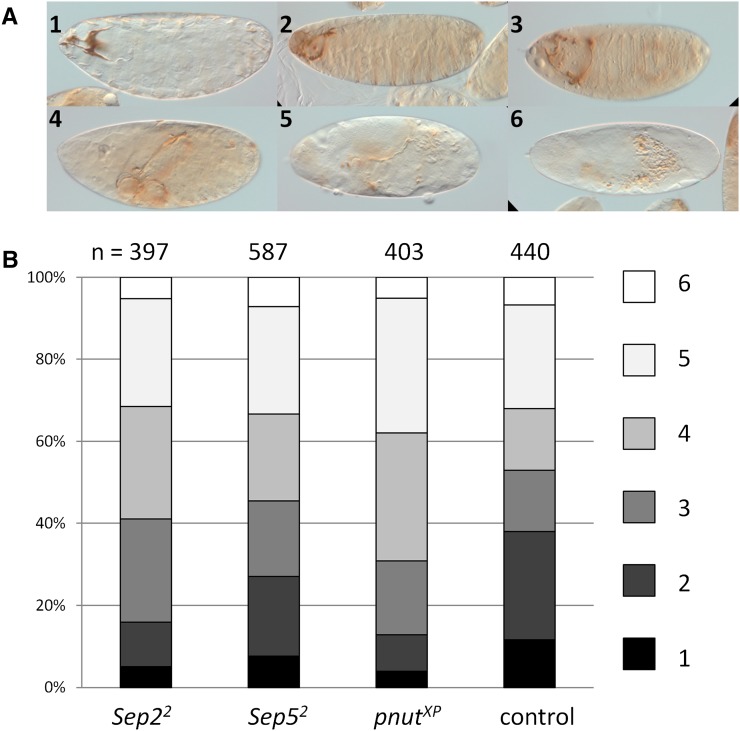
Septins enhance the *baz^4^* embryonic cuticle defect. (A) Embryonic cuticles were scored according to six categories; 1 is the least severe with wild-type cuticle and 6 is the most severe with only scraps of cuticle. (B) Zygotic *baz^4^* males are expected to be embryonic lethal and have varying degrees of defective cuticle (control). When the mother is heterozygous for *Sep2^2^*, *Sep5^2^*, or *pnut^XP^*, the cuticle defect of zygotic *baz^4^* males is more severe (Mann-Whitney *U*-test p-values for each mutant *vs.* control are 0.000217, 0.0129, and 2.28E-08, respectively). *Sep2^2^* and *Sep5^2^* are not significantly different from each other in their enhancement of *baz^4^* (p-value = 0.158), whereas *pnut^XP^* has a more severe enhancement compared to *Sep2^2^* (p-value = 0.011) or *Sep5^2^* (p-value = 0.000179).

*Baz* encodes a scaffolding protein important for cell polarity (Macara 2004; Margolis and Borg 2005; Wang and Margolis 2007). *Baz^4^* hemizygotes are embryonic lethal and have cuticle defects (Bilder *et al.* 2003). A screen for enhancers of this *baz^4^* phenotype identified *Sep5* (Shao *et al.* 2010). Here, we also find that the *baz^4^* embryonic cuticle phenotype is significantly enhanced in *Sep5^2^*, *Sep2^2^*, and *pnut^XP^* heterozygotes compared to *baz^4^* alone (Figure 5). The extent of enhancement by *Sep2^2^* or *Sep5^2^* is not significantly different, whereas the enhancement by *pnut^XP^* is significantly greater than *Sep2^2^* and *Sep5^2^*. To ask whether septins are required for the establishment or maintenance of cell polarity, we investigated the localization of several markers for cell polarity in *Sep5^2^*; *Sep2^2^* follicle cells, finding that the cell polarity components Armadillo and Discs-large and the cytoskeletal component α-spectrin localize correctly, and that *Sep2^2^* mutant follicle cells have wild-type localization of Baz-GFP (Figure S4). 

## Discussion

### Sep2 and Sep5 are redundant for Pnut localization

We found that Pnut fails to localize in *Sep5^2^*; *Sep2^2^* cells (Figure 4, F and G). This suggests that both Sep2 and Sep5 interact with Pnut by occupying the SEPT6 positions of the *D. melanogaster* septin complex. *Sep2^2^* mutants had lower levels of Pnut immunostaining at germline ring canals and follicle cells compared to wild-type or *hsFLP*; *Sep5^2^*; *FRT P{Ubi-GFP}/FRT Sep2^2^* cells, consistent with the higher expression level of *Sep2* compared to *Sep5* in oogenesis (modENCODE Anatomy RNA-seq; Attrill *et al.* 2016). In mammals, SEPT2 and SEPT6 subgroup members initiate complex assembly, followed by binding of SEPT7 (Sellin *et al.* 2011a); our results are consistent with this model of assembly, where the *D. melanogaster* SEPT7 subgroup member Pnut can only bind after the initiating septins, which presumably include Sep2 or Sep5, have assembled. Requirement of one septin for the localization of another has been found previously, including tissue-specific requirements; for example, in embryos *pnut* is required for localization of Sep1 (Fares *et al.* 1995) but not Sep2 (Adam *et al.* 2000), whereas in dorsal pupal epithelial cells *pnut* is required for localization of Sep2 (Founounou *et al.* 2013). 

### Septins are dispensable in germline

All five *D. melanogaster* septins localize to ring canals (Figure 3, Figure 4, and Figure S3), and *Sep2^2^* egg chambers show numbers of nurse cells suggestive of occasional defects in cystoblast division (O’Neill and Clark 2013). Consistent with previous observations for *pnut* mutant germline clones (Adam *et al.* 2000), we also found that *Sep5^2^*; *Sep2^2^* female germline clones are relatively common and do not show cell number defects. *Sep5^2^*; *Sep2^2^* germline cysts can fully develop into eggs, although we did not determine if these eggs are viable. Together, these observations suggest that septins have a subtle or nonessential role in the development of female germline cysts.

Septin localization at cytokinetic furrows and ring canals was detected previously. Cytokinetic furrow localization was observed for Sep1, Sep2, and Pnut in *Drosophila* S2 cells (Longtine *et al.* 1996; D’Avino *et al.* 2008), for Pnut and Sep2-GFP in pupal dorsal epithelium (Founounou *et al.* 2013), Pnut-mCherry in embryonic epithelium (Guillot and Lecuit 2013), and for Sep2-GFP in follicle cells (Morais-de-Sá and Sunkel 2013). Sep1, Sep2, and Pnut were all previously detected in germline ring canals during spermatogenesis (Hime *et al.* 1996). The presence of septins at germline ring canals during oogenesis was not entirely clear; concentrations of Sep2, Pnut, and Sep1 were sometimes observed at female germline ring canals (Fares *et al.* 1995; Adam 1999). The double ring localization of septin-GFPs is similar to the localization of phospho-tyrosine immunostaining found at male germline ring canals (Eikenes *et al.* 2015), and is reminiscent of the double septin ring at the bud neck of *S. cerevisiae* (Jiménez *et al.* 1998; Renz *et al.* 2016) and the double ring localization of Hts-RC in this study (Figure S1, F and G); thus, double ring localization may be an aspect of germline ring canal structure and a function that warrants further investigation. Although septins are not required for ring canal formation in the female germline, it is conceivable they might have a nonessential role at their outer rims, perhaps by maintaining membrane shape or rigidity (Tanaka-Takiguchi *et al.* 2009; Tooley *et al.* 2009; Mostowy *et al.* 2011; Gilden *et al.* 2012), regulating membrane remodelling (Sellin *et al.* 2011b), acting as a lateral diffusion barrier (Schmidt and Nichols 2004; Caudron and Barral 2009; Hu *et al.* 2010; Kwitny *et al.* 2010; Spiliotis and Gladfelter 2012; Clay *et al.* 2014; Ewers *et al.* 2014), or protein scaffolding (Hanrahan and Snyder 2003; Kozubowski *et al.* 2005; Kinoshita 2006; Hagiwara *et al.* 2011; Hall and Russell 2012; Feng *et al.* 2015). Although septins can alter microtubule organization (Kulic *et al.* 2008; Spiliotis 2010), and act as scaffolding for posttranslational modifiers of microtubules and motor proteins (Kremer *et al.* 2005; Spiliotis *et al.* 2008; Wloga and Gaertig 2010), we found that Orb accumulation, which is a microtubule-dependent process (Huynh and Johnston 2000), occurs normally in *Sep5^2^*; *Sep2^2^* oocytes, suggesting that transport via microtubules (at least in early stage egg chambers) is not septin-dependent. 

### Sep2 and Sep5 are required in follicle cells

Consistent with previous results (O’Neill and Clark 2013), the *Sep2^2^* mutants have egg chambers with abnormal numbers of nurse cells (Figure 1). Here, we determined that the majority of these were fused egg chambers where multiple germline cysts were encapsulated by follicle cells to form a single egg chamber. Although the *Sep2^2^* flies are *Sep5^+^*, the expression level and pattern of *Sep5* in oogenesis is not known. Overexpression of a *Sep5* cDNA transgene did not rescue the *Sep2^2^* egg chamber phenotype, whereas the *Sep2* cDNA did, thus showing that the function of Sep2 in oogenesis is unique and not redundant with Sep5. This contrasts with the observation that overexpressing *Sep5* rescues *Sep2^2^* male sterility, which shows that Sep2 and Sep5 proteins are redundant for male fertility. Wild-type *Sep5* expression is normally low in testes compared to *Sep2* which is moderately high (Chintapalli *et al.* 2007; Attrill *et al.* 2016), so *Sep2^2^* male sterility is probably due to loss of septin complex function via reduced expression of SEPT6 septins generally rather than *Sep2* specifically.

*Sep5^2^*; *Sep2^2^* follicle cell clones are typically small, and egg chambers with only double mutant follicle cells have reduced follicle cell numbers (Figure 2, C and E and Table 1). Further, double mutant follicle cells often have pyknotic nuclei, indicating cell death, or enlarged nuclei, indicating cytokinesis failure (Figure 2, C and D). These results suggest that *Sep2* and *Sep5* are redundant for follicle cell proliferation and maintenance. Consistent with this result, *pnut* is required for cytokinesis in follicle cells (Morais-de-Sá and Sunkel 2013). Further, this is reminiscent of the loss of imaginal cell proliferation in *Sep5*; *Sep2* double mutants and mutant clones (O’Neill and Clark 2013; Figure 2A) and *pnut^XP^* mutants (Neufeld and Rubin 1994), and the requirement for *pnut* in planar cell division of the pupal dorsal epithelium (Founounou *et al.* 2013) and actomyosin ring formation and constriction in embryonic epithelium (Guillot and Lecuit 2013). The irregular shape of *Sep5^2^*; *Sep2^2^* follicle cells may simply be due to a failure to proliferate and consequent stretching of cells as egg chambers grow, or it could represent a loss of apicobasal polarity in addition to proliferation defects. In mammalian epithelial cells, SEPT2 depletion leads to fibroblast-like cell shape and lack of polarity (Spiliotis *et al.* 2008). Although septins are implicated in the development and extension of cellular processes (Finger *et al.* 2003; Shinoda *et al.* 2010) and the coordination of cell movements (Chacko *et al.* 2005), *Sep5^2^*; *Sep2^2^* follicle cells are able to envelope germline cysts, suggesting that septins are not required for the formation of the cellular processes and the migration required for the encapsulation of germline cysts. Thus, it appears that septins are required for specific types of cell division in *D. melanogaster*, as they are in mammals (Menon *et al.* 2014), including epithelial cell divisions (Founounou *et al.* 2013). Further, we found that *Sep2* mutant egg morphology is independent of the genotype of germline, suggesting that mutant follicle cells are responsible; thus, it is possible that septins play a role in follicle cell rotation during oogenesis, which is required for egg elongation (Haigo and Bilder 2011). 

### Punctate septins

Punctate or ring-like cytoplasmic localization of septins (Figure 3, Figure 4, and Figure S3) has also been previously reported. In *D. melanogaster*, apically distributed puncta of Sep2-GFP, Sep5-GFP, and Pnut were observed in epithelia (Founounou *et al.* 2013), and all septin-GFP fusions localized as puncta during cellularization of blastoderm embryos (Su *et al.* 2013). In human K562 cells, septin discs of ∼0.8 µm diameter were observed during interphase; these discs were dependent on microtubules, and were disrupted during fixation (Sellin *et al.* 2011b). Larger and more prominent puncta were observed for *nosGAL4* driven septin-GFPs compared to Sep2-GFP and Pnut immunostaining, suggesting that overexpression can lead to septin-GFP aggregation. However, Pnut immunostaining does have a punctate appearance, indicating that septin puncta are not entirely artifactual. It is not clear whether these puncta have a specific cytoplasmic function, such as cytoplasmic cytoskeletal organization, or if they act as a septin reserve that can be rapidly deployed at the onset of cytokinesis or membrane deformation. 

### A link between cell polarity and septin function

*Baz* encodes a scaffolding protein that is important for cell polarity (Macara 2004; Margolis and Borg 2005; Wang and Margolis 2007); for example, in the *D. melanogaster* embryonic epithelium, Baz is apically localized, is required to establish apicobasal polarity, and forms complexes with Par-6/aPKC and cadherin (Harris and Peifer 2004, 2005). While a previous screen found that the only septin to enhance the mutant *baz* embryonic cuticle defect was *Sep5* (Shao *et al.* 2010), we found that at least three septins, *Sep2*, *Sep5*, and *pnut*, are enhancers. The lower level of *baz^4^* enhancement by *Sep2^2^* and *Sep5^2^* is consistent with redundancy of *Sep2* and *Sep5*, both SEPT6 septins, compared to *pnut*, which is the only SEPT7 septin in *D. melanogaster*. 

The enhancement of the *baz^4^* embryonic cuticle phenotype by septin mutants (Figure 5), the irregular shape and frequent loss of epithelial structure of *Sep5^2^*; *Sep2^2^* follicle cells (Figure 2C and Table 1), and polarized localization of *D. melanogaster* septins found here (Figure 4B) and by others (Shao *et al.* 2010; Founounou *et al.* 2013) suggest a link between septins and cell polarity in flies. In mammalian epithelial cells, septins associate with specific microtubule tracks to facilitate polarized transport of vesicles, and septin depletion leads to loss of apical and basolateral membrane protein localization (Spiliotis *et al.* 2008). We reasoned that septins might be involved in the establishment or maintenance of cell polarity in *D. melanogaster* epithelial cells. However, whereas *Sep5^2^*; *Sep2^2^* clones had disrupted localization of Pnut, several components of cell polarity pathways localized normally, suggesting that cell polarity does not require septins, and that septins function downstream of cell polarity or in an independent pathway. Potential connections between Baz and septins should be further explored. Baz interacts with the lipid phosphatase PTEN to regulate actin cytoskeleton organization and generate an apical enrichment of phosphatidylinositol (4,5) bisphosphate (von Stein *et al.* 2005). In humans, phosphatidylinositol (4,5) bisphosphate is bound by SEPT4 and required for septin filament formation (Zhang *et al.* 1999). So, it is possible that septin localization and function depend on proper localization of Baz. 

### Conclusions

The relationship between septin complex assembly and the diversity of the various septin subgroups raises the intriguing idea that the functional characteristics of individual septin complexes depend on their subunit composition, thus providing a mechanism to allow multiple distinct populations of septin complexes to operate independently within a single cell (Kinoshita 2006; Cao *et al.* 2009; Hernández-Rodríguez *et al.* 2014). Our investigation of *Sep2* and *Sep5* finds that they are redundant in imaginal tissues and follicle cells, yet *Sep2* maintains a unique function in follicle cells. Thus, assuming that *Sep2* ancestral function is conserved, we suggest that, after *Sep5* arose via retroduplication, it underwent partial loss of this ancestral protein function. Whether the diversification of *Sep2* and *Sep5* is representative of septin evolution generally, and of septin functional diversity in other lineages, such as mammals with 13 septin genes, is unclear; it is worth noting that most human septins arose before the divergence of tetrapods from fish (Cao *et al.* 2007) and have thus had significantly more time for functional diversification than *Sep2* and *Sep5*. This work highlights the importance of considering subgroup member redundancy and the potential diversity of functions across tissues when studying animal septins. 

## Supplementary Material

Supplemental Material
