# Supplementary material for: Partial Functional Diversification of Drosophila melanogaster Septin Genes Sep2 and Sep5
Source: G3 (Bethesda). 2016 May 2;6(7):1947–57. doi: 10.1534/g3.116.028886 (PMC4938648; doi:10.1534/g3.116.028886)
Supplement: Supplemental Material [file supp_6_7_1947__index.html]

Partial Functional Diversification of Drosophila melanogaster Septin Genes Sep2 and Sep5 — Supplemental Material 

# Partial Functional Diversification of *Drosophila melanogaster* Septin Genes *Sep2* and *Sep5*

## Supplemental Material for O'Neill and Clark, 2016

**Files in this Data Supplement:**

- Figure S1 - *Sep22 Sep52* double mutant germline cysts have wild-type distribution of several proteins. (.pdf, 389 KB)
- Figure S2 - *Sep2-GFP* and *Sep5-GFP* fusion proteins are functional. (.pdf, 347 KB)
- Figure S3 - Sep1-GFP and Sep4-GFP localization in oogenesis. (.pdf, 550 KB)
- Figure S4 - *Sep22 Sep52* double mutant follicle cells have wild-type distribution of several cell polarity proteins. (.pdf, 665 KB)
