# Supplementary material for: Partial Functional Diversification of Drosophila melanogaster Septin Genes Sep2 and Sep5
Source: G3 (Bethesda). 2016 May 2;6(7):1947–57. doi: 10.1534/g3.116.028886 (PMC4938648; doi:10.1534/g3.116.028886)
Supplement: Supplemental Material [file supp_g3.116.028886_FigureS1.pdf]

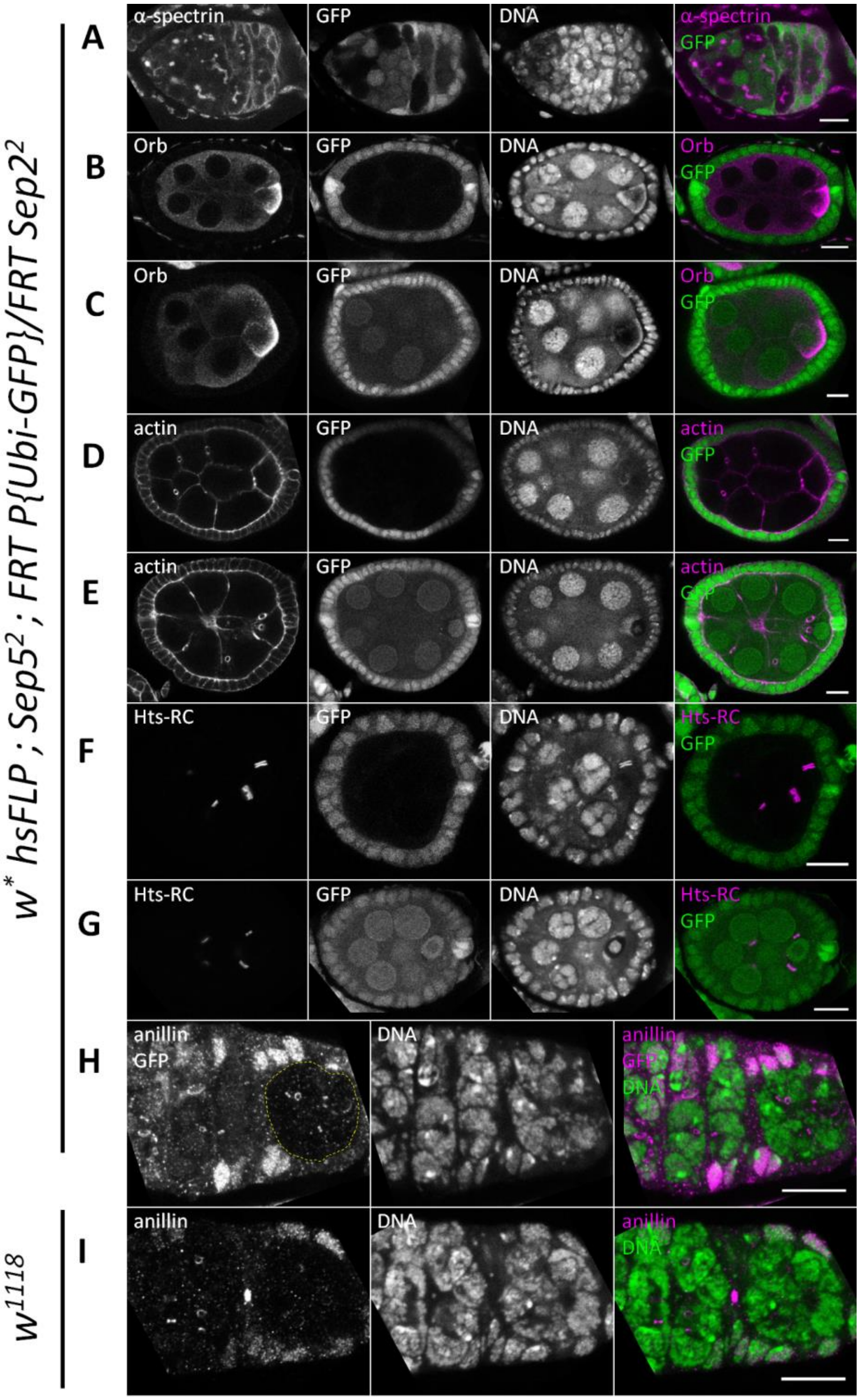

Figure S1 – *Sep2²* *Sep5²* double mutant germline cysts have wild-type distribution of several proteins.

w hsFLP; Sep5²; FRT P{Ubi-GFP}/FRT Sep2²* was used to generate double mutant clones. Distribution of several proteins was compared between *Sep5²; Sep2²* double mutant (GFP negative) and *Sep5²; Sep2²/Ubi-GFP* and *Sep5²; Ubi-GFP* (GFP positive) germline cysts: α-spectrin (A; fusome), Orb (B, C; oocyte), actin (D, E; cell cortex, ring canals), Hts-RC (F, G; ring canals). Distribution of anillin (H, I; cytokinetic furrow, early ring canals) was also compared to *w¹¹¹⁸*, since anillin and GFP were collected in the same channel. Dotted line highlights a double mutant cyst in H. In all cases, the protein did not appear to be disrupted in double mutant cells. Scale bar = 10 μm.
