# Supplementary material for: Partial Functional Diversification of Drosophila melanogaster Septin Genes Sep2 and Sep5
Source: G3 (Bethesda). 2016 May 2;6(7):1947–57. doi: 10.1534/g3.116.028886 (PMC4938648; doi:10.1534/g3.116.028886)
Supplement: Supplemental Material [file supp_g3.116.028886_FigureS2.pdf]

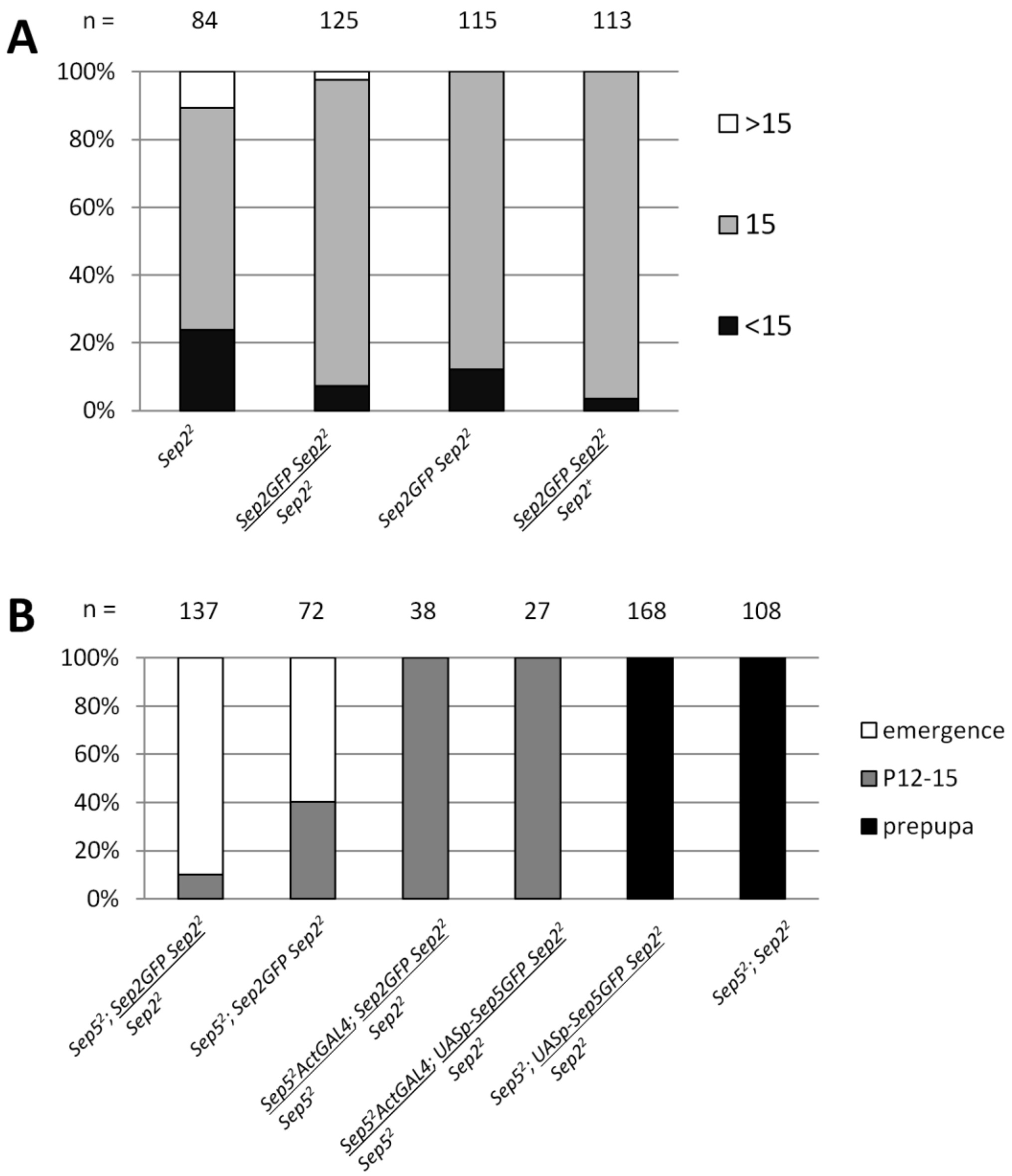

Figure S2 – *Sep2-GFP* and *Sep5-GFP* fusion proteins are functional.
A. *Sep2*^{2} egg chambers often have more or fewer than 15 nurse cells. *Sep2-GFP* rescues the *Sep2*^{2} egg chamber phenotype in a dosage dependent manner. *Sep2-GFP Sep2*^{2}/*Sep2*$^+$ have occasional egg chambers with 13-14 nurse cells, suggesting a minor dominant negative effect of *Sep2-GFP* on cystoblast divisions. B. *Sep2-GFP* rescues double mutant prepupal lethality, although having two copies of *Sep2-GFP* increases the proportion of individuals that arrest at the end of metamorphosis, suggesting a minor dominant negative effect of the transgene. *Sep5-GFP* also rescues the double mutant prepupal lethality.
