# Supplementary material for: Partial Functional Diversification of Drosophila melanogaster Septin Genes Sep2 and Sep5
Source: G3 (Bethesda). 2016 May 2;6(7):1947–57. doi: 10.1534/g3.116.028886 (PMC4938648; doi:10.1534/g3.116.028886)
Supplement: Supplemental Material [file supp_g3.116.028886_FigureS3.pdf]

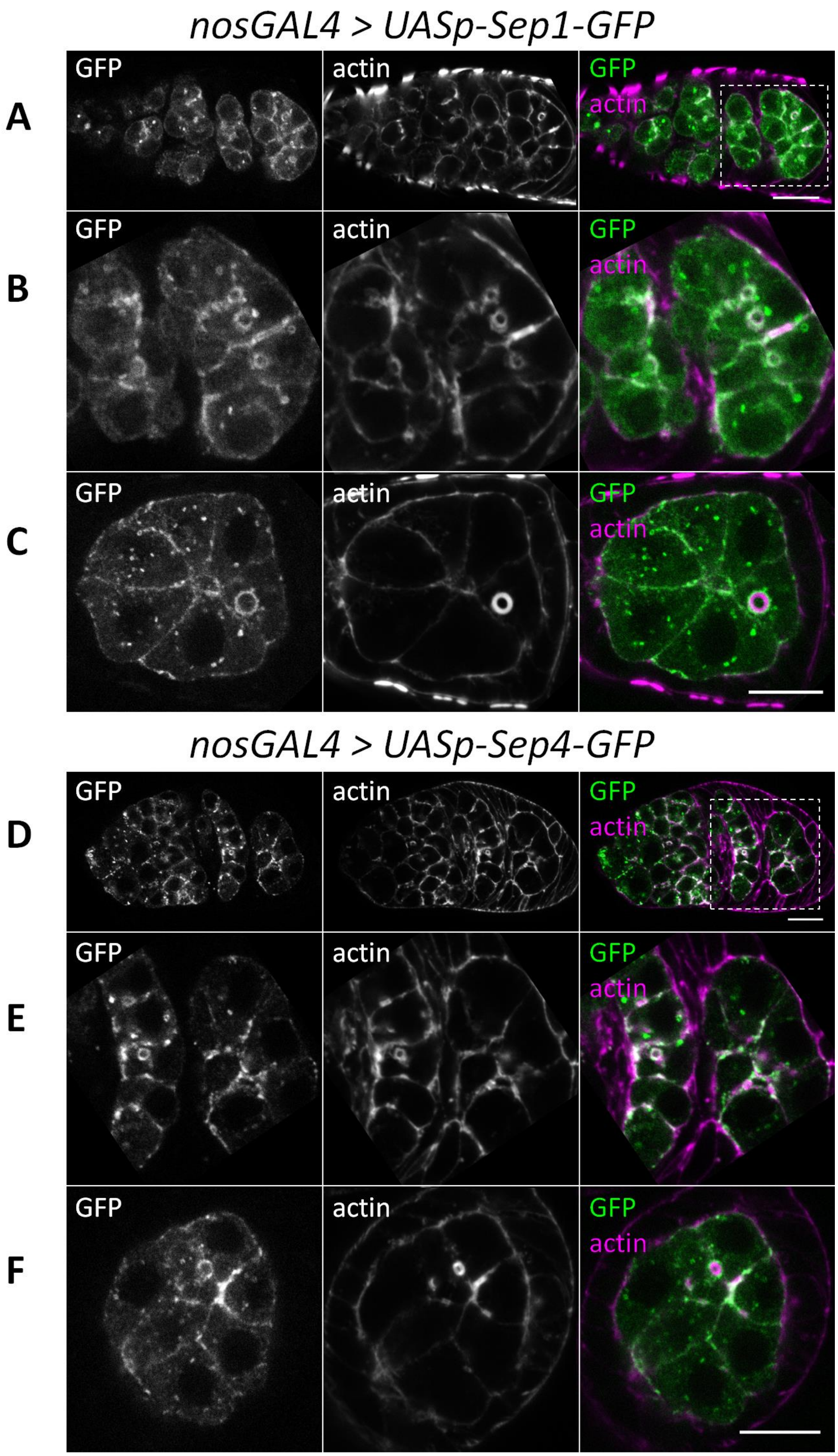

Figure S3 – Sep1-GFP and Sep4-GFP localization in oogenesis. *nosGAL4* was used to drive *UASp-Sep1-GFP* (A, B, and C) and *UASp-Sep4-GFP* (D, E, and F) transgenes in oogenesis. Both Sep1-GFP and Sep4-GFP localize cytoplasmically with a concentration at the cell cortex in germline cysts (A, D), to the outer rim of ring canals and as cytoplasmic punctae (B, C, E, F). Scale bar = 10 μm.
