# Supplementary material for: Partial Functional Diversification of Drosophila melanogaster Septin Genes Sep2 and Sep5
Source: G3 (Bethesda). 2016 May 2;6(7):1947–57. doi: 10.1534/g3.116.028886 (PMC4938648; doi:10.1534/g3.116.028886)
Supplement: Supplemental Material [file supp_g3.116.028886_FigureS4.pdf]

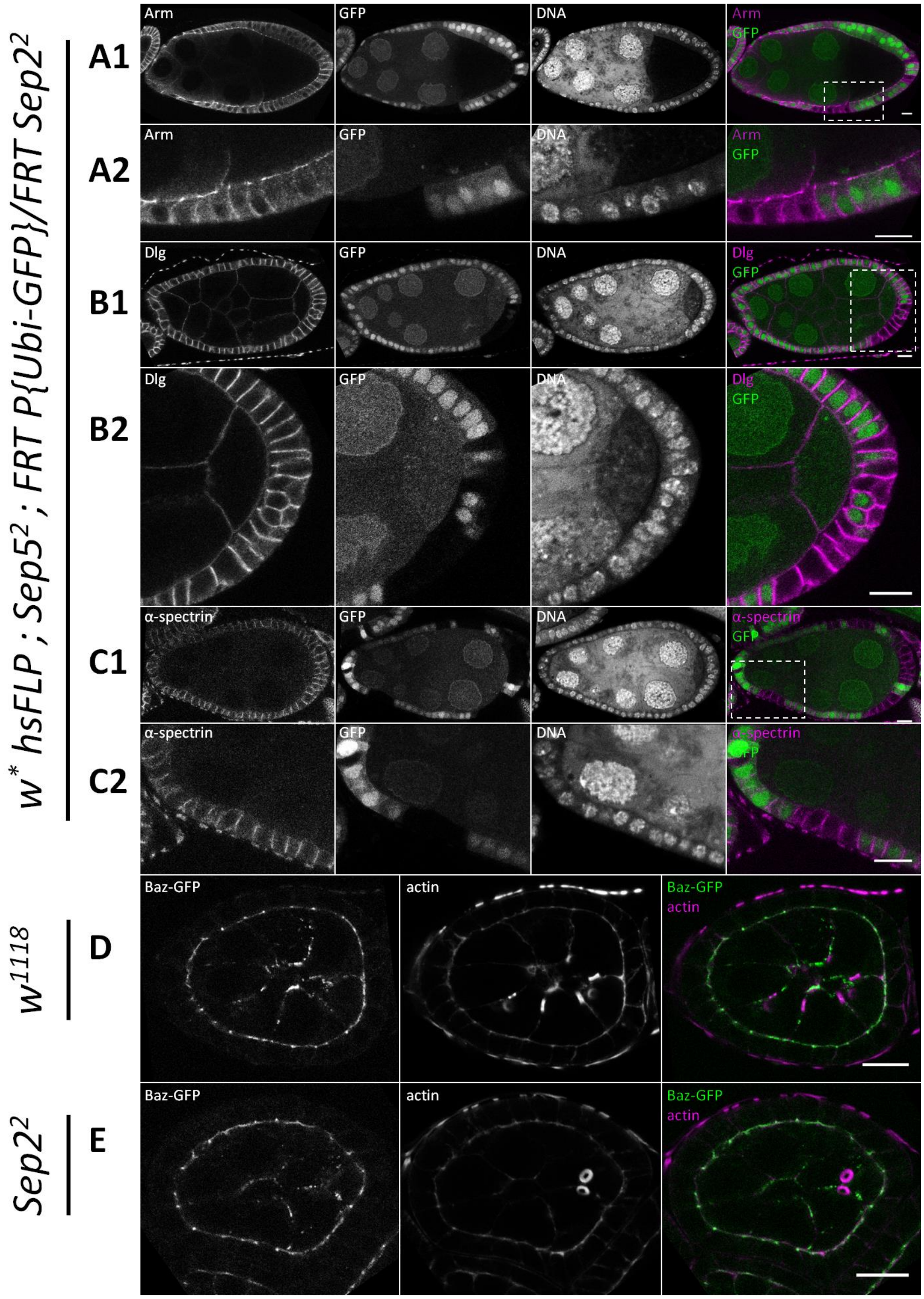

Figure S4 – *$Sep2^2$ $Sep5^2$* double mutant follicle cells have wild-type distribution of several cell polarity proteins.

w hsFLP; $Sep5^2$; FRT P{His-GFP}/FRT $Sep2^2$* was used to generate double mutant clones. Distribution of several proteins were compared between *$Sep5^2$; $Sep2^2$* double mutant (GFP negative) and *$Sep5^2$; $Sep2^2$/His-GFP* and *$Sep5^2$; His-GFP* (GFP positive) follicle cells: armadillo (A1, A2; Arm, cell polarity), Discs large (B1, B2; cell polarity), and α-spectrin (C1, C2; cytoskeleton). White squares are shown magnified in the subsequent descending panels. Distribution of Baz-GFP was compared between *w^{1118}* (D) and *$Sep2^2$* (E). In all cases, the marker tested did not appear to be disrupted in double mutant cells. Scale bar = 10 μm.
